# Online COVID-19 diagnosis prediction using complete blood count: an innovative tool for public health

**DOI:** 10.1186/s12889-023-17477-8

**Published:** 2023-12-19

**Authors:** Xiaojing Teng, Zhiyi Wang

**Affiliations:** 1 Department of Clinical Laboratory, Affiliated Hangzhou First People’s Hospital, Westlake University School of Medicine, Hangzhou, Zhejiang 310000 China; 2 Department of Clinical Laboratory, Hangzhou Women’s Hospital (Hangzhou Maternity and Child Health Care Hospital), No. 369, Kunpeng Road, Shangcheng District Hangzhou, Hangzhou, Zhejiang 310008 China

**Keywords:** COVID-19, Machine learning, XGBoost, Complete blood Count (CBC)

## Abstract

**Background:**

COVID-19, caused by SARS-CoV-2, presents distinct diagnostic challenges due to its wide range of clinical manifestations and the overlapping symptoms with other common respiratory diseases. This study focuses on addressing these difficulties by employing machine learning (ML) methodologies, particularly the XGBoost algorithm, to utilize Complete Blood Count (CBC) parameters for predictive analysis.

**Methods:**

We performed a retrospective study involving 2114 COVID-19 patients treated between December 2022 and January 2023 at our healthcare facility. These patients were classified into fever (1057 patients) and pneumonia groups (1057 patients), based on their clinical symptoms. The CBC data were utilized to create predictive models, with model performance evaluated through metrics like Area Under the Receiver Operating Characteristics Curve (AUC), accuracy, sensitivity, specificity, and precision. We selected the top 10 predictive variables based on their significance in disease prediction. The data were then split into a training set (70% of patients) and a validation set (30% of patients) for model validation.

**Results:**

We identified 31 indicators with significant disparities. The XGBoost model outperformed others, with an AUC of 0.920 and high precision, sensitivity, specificity, and accuracy. The top 10 features (Age, Monocyte%, Mean Platelet Volume, Lymphocyte%, SIRI, Eosinophil count, Platelet count, Hemoglobin, Platelet Distribution Width, and Neutrophil count.) were crucial in constructing a more precise predictive model. The model demonstrated strong performance on both training (AUC = 0.977) and validation (AUC = 0.912) datasets, validated by decision curve analysis and calibration curve.

**Conclusion:**

ML models that incorporate CBC parameters offer an innovative and effective tool for data analysis in COVID-19. They potentially enhance diagnostic accuracy and the efficacy of therapeutic interventions, ultimately contributing to a reduction in the mortality rate of this infectious disease.

**Supplementary Information:**

The online version contains supplementary material available at 10.1186/s12889-023-17477-8.

## Background

 The novel coronavirus (SARS-CoV-2), first identified in Wuhan, Hubei Province, China, in 2019, subsequently triggered a global pandemic. The virus primarily spreads through respiratory droplets and aerosols, while contact with contaminated objects can also potentially lead to infection. Post-infection, patients may exhibit diverse symptoms like fever due to the immune response to the virus. The severity and duration of these symptoms vary widely among individuals, potentially progressing into a lung infection known as COVID-19. The progression of COVID-19 involves multiple factors, such as individual immunity, underlying diseases, epidemiological history, exposure dose, and more [1]. Delayed or inadequate treatment of COVID-19 may escalate into critical complications, including acute respiratory distress syndrome (ARDS), multiple organ failure, thrombosis, and other potentially fatal conditions.

COVID-19 diagnosis largely depends on chest imaging; however, its utility is often limited in healthcare centers and community hospitals due to constraints such as lack of professional equipment, disparities in technical expertise, and radiation risks. Furthermore, the clinical manifestations and radiographic features of COVID-19 have many similarities with other respiratory infections. Further, the clinical manifestations and radiographic features of COVID-19 share many similarities with other respiratory infections, which do not fully reflect the severity and prognosis of the disease, nor rule out other potential diagnoses [2]. As such, there is an urgent need for a novel, portable, and rapid predictive tool for broader application in clinical settings.

Complete Blood Count (CBC), a routine and cost-effective blood test, provides vital information about various blood parameters. Recent years have seen the introduction of new indicators, such as the Neutrophil to Lymphocyte Ratio (NLR), derived NLR (dNLR), Platelet to Lymphocyte Ratio (PLR), Monocyte to Lymphocyte Ratio (MLR), and Systemic Inflammatory Response Index (SII), proposed as biomarkers for assisting diagnosis, assessing disease progression, and evaluating risk [3, 4]. However, studies leveraging these indicators for COVID-19 prediction remain limited. Artificial intelligence (AI) and machine learning are increasingly employed across various fields, with significant innovations in disease prediction, including cardiovascular diseases, neurodegenerative disease [5], cancer [6], neurodegenerative diseases [7], and infectious diseases [8]. AI can provide relatively accurate and reliable predictions based on diverse data sources and models.

In this study, we seek to integrate CBC parameters with machine learning to construct an online predictive model for COVID-19, aiming to enhance the efficiency of diagnosis and treatment for this global health concern.

## Methods

### Study population

In this retrospective study, we analyzed a cohort of 2561 patients with confirmed COVID-19, who visited our clinic between December 2022 and January 2023. All cases were confirmed by reverse transcription polymerase chain reaction (RT-PCR) for SARS-CoV-2. Based on the inclusion and exclusion criteria, a total of 2114 COVID-19 patients were included for analysis. The patients were stratified into two groups according to clinical manifestations: the fever group (1057 cases) and the pneumonia group (1057 cases). This study received approval from the Ethics Committee of the First People’s Hospital of Hangzhou (Ethics Approval Number ZN2023018).

We also collected an external validation cohort of 513 patients with a confirmed diagnosis of COVID-19 who presented to the same institution for the first time between December 2022 and January 2023. These patients were included for external validation purposes, undergoing the same treatment protocols as those applied in the initial cohort for comparative analysis and assessment.

Inclusion Criteria: Patients aged ≥ 18 years, exhibiting clinical symptoms such as fever, cough, sputum production, difficulty breathing, chest pain, fatigue, muscle or body aches, headache, new loss of taste or smell, sore throat, congestion or runny nose, nausea or vomiting, and diarrhea. Evidence of new or progressive pulmonary consolidation or infiltration on chest X-ray or CT scan was required, along with RT-PCR confirmed SARS-CoV-2 positivity. Evidence of new or progressive pulmonary consolidation or infiltration on chest X-ray or CT scan was required, along with RT-PCR confirmed SARS-CoV-2 positivity.

Exclusion Criteria: Presence of other severe or unstable functional organ abnormalities or systemic diseases (e.g., heart failure, liver cirrhosis, renal failure, malignant tumors, etc.); known allergy or contraindication to certain medications (such as allergies to penicillin or macrolides, or the use of CYP3A4 inducers or inhibitors); receipt of other therapeutic anti-infective drugs within 24 h before enrollment, such as antibacterial drugs, or the use of antifungal or antiparasitic drugs within 72 h before enrollment.

### Data collection

All patient data was obtained from the Hospital Information System (HIS). This included demographic information such as age and gender, as well as clinical diagnoses. Additionally, laboratory tests were conducted on all patients, with data extracted from the Laboratory Information System (LIS). These tests comprised White Blood Cells (WBC), Neutrophil percentage (Neu.%), Lymphocyte percentage (Lym.%), Monocyte percentage (Mon.%), Eosinophil percentage (Eos.%), Basophil percentage (Bas.%), Neutrophil count (Neu.#), Lymphocyte count (Lym.#), Monocyte count (Mon.#), Eosinophil count (Eos.#), Basophil count (Bas.#), Hemoglobin (HGB), Red Blood Cells (RBC), Hematocrit (HCT), Mean Corpuscular Volume (MCV), Mean Corpuscular Hemoglobin (MCH), Mean Corpuscular Hemoglobin Concentration (MCHC), Red Cell Distribution Width (RDW-cv), Platelet count (PLT), Plateletcrit (PCT), Mean Platelet Volume (MPV), Platelet Distribution Width (PDW), and high-sensitivity C-Reactive Protein (hs-CRP). All blood samples were processed using the BC-7500 Automatic Hematology Analyzer (Mindray) using the manufacturer’s reagent kits. Our laboratory ensured the quality of results through regular internal quality control and required external quality assessments.

### Derived inflammatory indices

We derived several inflammation indices based on the collected cell counts, as follows:

1. Systemic Inflammation Index (SII): Neutrophil **╳** Platelet / Lymphocyte
2. Systemic Inflammation Response Index (SIRI): Neutrophil **╳** Monocyte / Lymphocyte
3. Aggregated Inflammation Index (AISI): Neutrophil **╳** Platelet **╳** Monocyte / Lymphocyte
4. Neutrophil to Lymphocyte Ratio (NLR): Neutrophil / Lymphocyte
5. Platelet to Lymphocyte Ratio (PLR): Platelet / Lymphocyte
6. Lymphocyte to Monocyte Ratio (LMR): Lymphocyte / Monocyte
7. NLPR: Neutrophil **╳** Lymphocyte **╳** Platelet
8. Derived Neutrophil to Lymphocyte Ratio (dNLR): (WBC - Lymphocyte) / Lymphocyte

These derived indices were used in our machine learning model to enhance the predictive ability for COVID-19 diagnosis.

### Model development using statistical analysis and machine learning

All statistical data were analyzed using R software on Windows and the Deepwise and Beckman Coulter DxAI platform (https://dxonline.deepwise.com). Categorical variables are represented as frequencies and percentages, and continuous variables are represented as mean ± standard deviation (SD) or median with interquartile range (IQR). Clinical features and complete blood count results were compared using Student’s t-test, Mann–Whitney test, or chi-square test. Variables associated with pneumonia were identified using Spearman correlation analysis, with *p* < 0.05 considered statistically significant.

The predictive model was constructed using the Deepwise and Beckman Coulter DxAI platform. Features were filtered using significance tests and correlation analysis. Firstly, significance tests were carried out to select the variables that are significantly different between the fever group and the pneumonia group. The statistical difference was calculated by Student’s t-test, Mann-Whitney test, or chi-square test with *P* < 0.05 was considered statistically significant. Then, we employed feature correlation analysis discarded feathers with a correlation coefficient below 0.9.

The initial step in feature selection involved computing the Pearson correlation coefficient (PCC) among all features to identify highly correlated pairs. Any pair with a PCC value exceeding 0.9 had one feature randomly removed to reduce redundancy and potential multicollinearity [9]. Using the selected features, four types of predictive models were constructed: Adaboost, XGBoost, Random Forest, and LogisticRegression. To further enhance the performance of these models, a refinement step was implemented by ranking the features according to their importance and selecting the top ten features from the best performing model. This ensures that the models are built on the most influential features, potentially improving their predictive accuracy.

Our dataset was split into a 70% training set and a 30% validation set for the evaluation of model performance. Model performance was assessed using Receiver Operating Characteristic (ROC) curves, with the Area Under the Curve (AUC) calculated for each model. An AUC value closer to 1.0 indicates stronger predictive power. Calibration curves were generated to evaluate the proximity of the model’s predicted risk to the actual risk, with Decision Curve Analysis (DCA) applied to assess the decision utility of each model.

## Results

### Comparison of clinical features

**Fig. 1:**
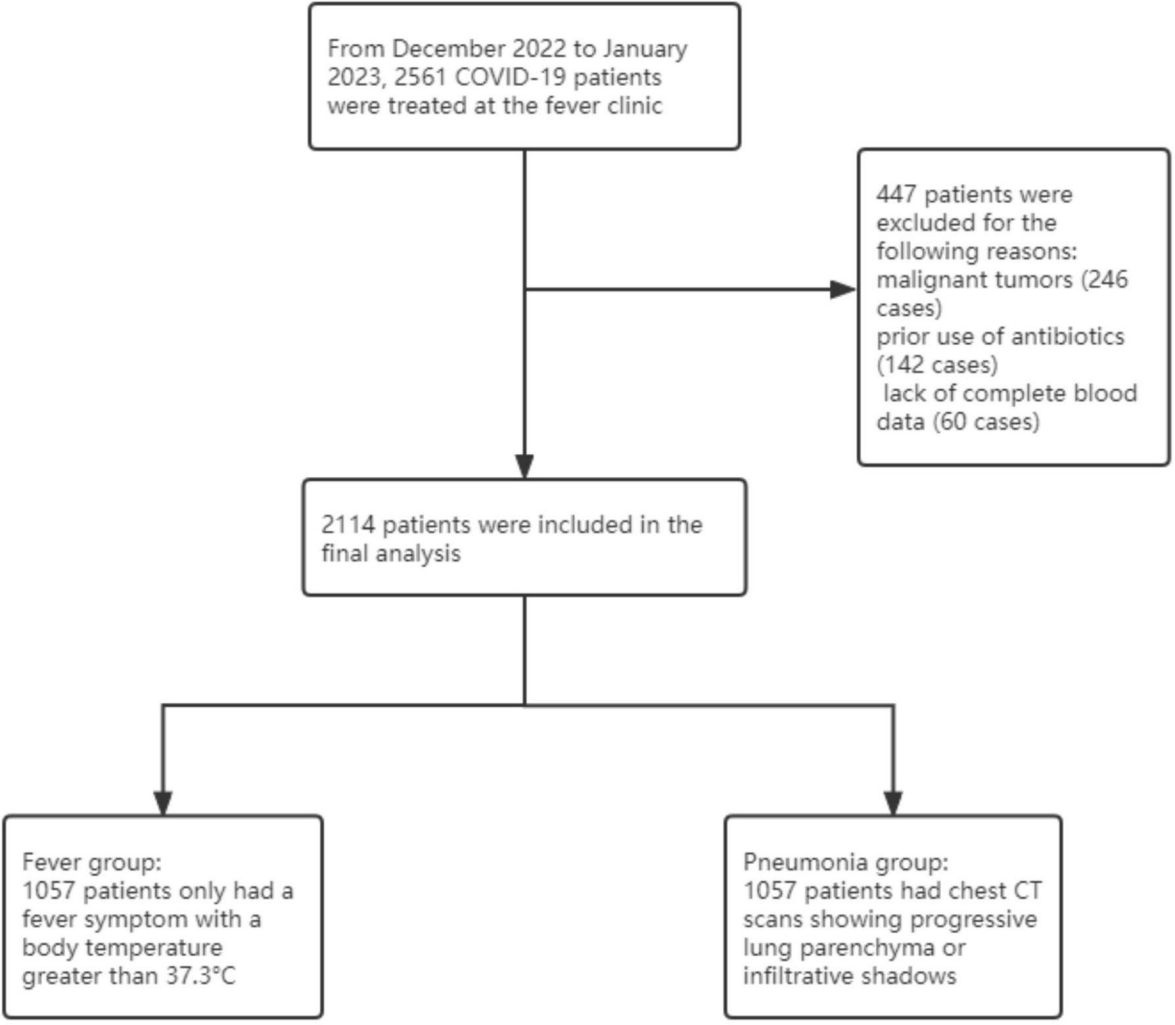
Study flow showing patients excluded from the study and the final cohort included in the study

This study retrospectively reviewed 2561 COVID-19 patients who visited the Fever Clinic of Hangzhou First People’s Hospital from December 2022 to January 2023. Following the screening based on inclusion and exclusion criteria, a total of 2114 COVID-19 patients were finally included. According to clinical characteristics, the patients were divided into a fever group (*n* = 1057) and a pneumonia group (*n* = 1057). The study workflow is depicted in Fig. 1.

There were no statistically significant differences between the two groups in terms of Hs-CRP and Bas.% (*p* > 0.05). The proportion of female patients was significantly higher than male patients in both groups (*p* < 0.001). Patients in the pneumonia group were significantly older than those in the fever group (Z = 628478.5, *p* < 0.001). In the fever group, the levels of AISI, SII, SIRI, NLR, PLR, dNLR, Neu.%, Mon.%, Neu.#, Mon.#, HGB, RBC, HCT, MCV, MCH, MCHC, MPV, and PDW were all significantly elevated compared to the pneumonia group, with each parameter showing a *P* value < 0.001, indicating strong statistical significance. Contrastingly, the Fever group displayed significantly lower levels of NLPR, LMR, Lym.%, Eos.%, Lym.#, Eos.#, PLT, and PCT compared to the Pneumonia group (all *P* < 0.001), except for WBC, where the difference was also significant but with a *P* value of 0.002, as shown in Table 1.

**Table 1:** Baseline clinical and biochemical characteristics of all patients

Variable	Group		*Z/x²*	*p-value*
	fever(*n* = 1057)	pneumonia(*n* = 1057)		
Age	31.00(23.00–56.000)	33.00(24.00–65.00)	628478.5	0.000^**^
Gender				0.000^**^
Female	673 (63.7%)	534 (50.5%)		
Male	384 (36.3%)	523 (49.5%)		
AISI	581.00(222.00-1238.57)	229.83(111.96-496.87)	763968.5	0.000^**^
SII	1033.46(476.00-2052.00)	486.53(261.04-928.33)	766510.0	0.000^**^
SIRI	3.04(1.28–6.08)	0.98(0.50–2.10)	819337.0	0.000^**^
NLPR	0.021(0.013–0.032)	0.029(0.019–0.046)	408272.0	0.000^**^
NLR	5.33(2.71–10.14)	2.04(1.15–4.11)	826663.5	0.000^**^
PLR	207.50(134.62–346.00)	126.36(89.38-181.43)	802169.5	0.000^**^
LMR	1.63(1.00-2.75)	3.75(2.33–5.75)	237065.0	0.000^**^
dNLR	1.67(1.40-2.00)	1.33(1.21–1.50)	854776.0	0.000^**^
WBC(╳10^9^ L)	6.40(4.90–8.40)	6.70(5.10-9.00)	515015.0	0.002^**^
Neu. %	75.30(64.60–83.00)	60.20(47.50–72.80)	801109.0	0.000^**^
Lym.%	14.00(8.00-23.70)	29.60(17.80–41.40)	285206.5	0.000^**^
Mon.%	8.50(6.50–10.90)	7.20(5.80–9.30)	684413.0	0.000^**^
Eos.%	0.30(0.10–0.90)	0.90(0.20–2.20)	399141.0	0.000^**^
Bas.%	0.20(0.10–0.30)	0.20(0.10–0.30)	536631.5	0.108
Neu.#(╳10^9^ L)	4.70(3.30–6.70)	3.80(2.50–5.60)	669622.5	0.000^**^
Lym.#(╳10^9^ L)	0.90(0.60–1.30)	1.80(1.10–2.90)	254022.0	0.000^**^
Mon.#(╳10^9^ L)	0.60(0.40–0.70)	0.50(0.40–0.70)	624082.0	0.000^**^
Eos.#(╳10^9^ L)	0.02(0.01–0.06)	0.06(0.01–0.15)	388349.0	0.000^**^
Bas.#(╳10^9^ L)	0.01(0.01–0.02)	0.01(0.01–0.02)	510839.0	0.000^**^
HGB (g/L)	140.00(129.00-152.00)	131.00(122.00-140.00)	735503.5	0.000^**^
RBC (╳10^9^ L)	4.64(4.31–5.04)	4.56(4.21–4.88)	628034.5	0.000^**^
HCT	0.42(0.39–0.45)	0.39(0.37–0.42)	722600.5	0.000^**^
MCV (fl.)	90.00(87.30–92.40)	87.20(83.20–91.10)	719023.5	0.000^**^
MCH (pg)	30.30(29.30–31.20)	29.10(27.50–30.50)	738029.0	0.000^**^
MCHC (g/L)	336.00(332.00-341.00)	334.00(328.00-339.00)	667044.5	0.000^**^
RDW-cv (%)	13.00(12.00–13.00)	13.00(13.00–14.00)	503687.5	0.000^**^
PLT (╳10^9^ L)	193.00(160.00-236.00)	233.00(173.00-302.00)	400241.0	0.000^**^
PCT	0.20(0.16–0.23)	0.22(0.17–0.28)	425123.0	0.000^**^
MPV (fl.)	10.10(9.40–10.80)	9.60(8.90–10.40)	683973.0	0.000^**^
PDW (fl.)	11.50(10.40–13.00)	10.85(9.70–12.60)	651739.0	0.000^**^
Hs-CRP	10.00(4.30-21.45)	11.40(2.60–33.60)	501992.5	0.253

*AISI *aggregate index of systemic inflammation (neutrophil ╳ platelet ╳ monocyte to lymphocyte ratio), *dNLR *derived neutrophil to lymphocyte ratio, *MLR *monocyte to lymphocyte ratio, *MPR *mean platelet volume to platelet ratio, *NLR *neutrophil to lymphocyte ratio, *NLPR *neutrophil to lymphocyte ╳ platelet ratio, *PLR *platelet to lymphocyte ratio, *SII *systemic immune-inflammation index (neutrophil ╳ platelet to lymphocyte ratio), *SIRI *systemic inflammation response index (neutrophil ╳ monocyte to lymphocyte ratio), *WBC *White blood cells, *HGB *Haemoglobin, *HCT *Hematocrit, *MCV *mean corpuscular volume, *MCH *mean corpuscular hemoglobin, *MCHC *mean corpuscular hemoglobin concentration, *RDW *Red blood cell distribution width, *PLT *Platelet, *PCT *Thrombocytocrit, *MPV *mean platelet volume, *PDW *platelet distribution width, *hs-CRP* hypersensitive C-reactive protein

^*^p < 0.05;

^**^p < 0.001

### Correlation analysis with COVID-19

**Fig. 2:**
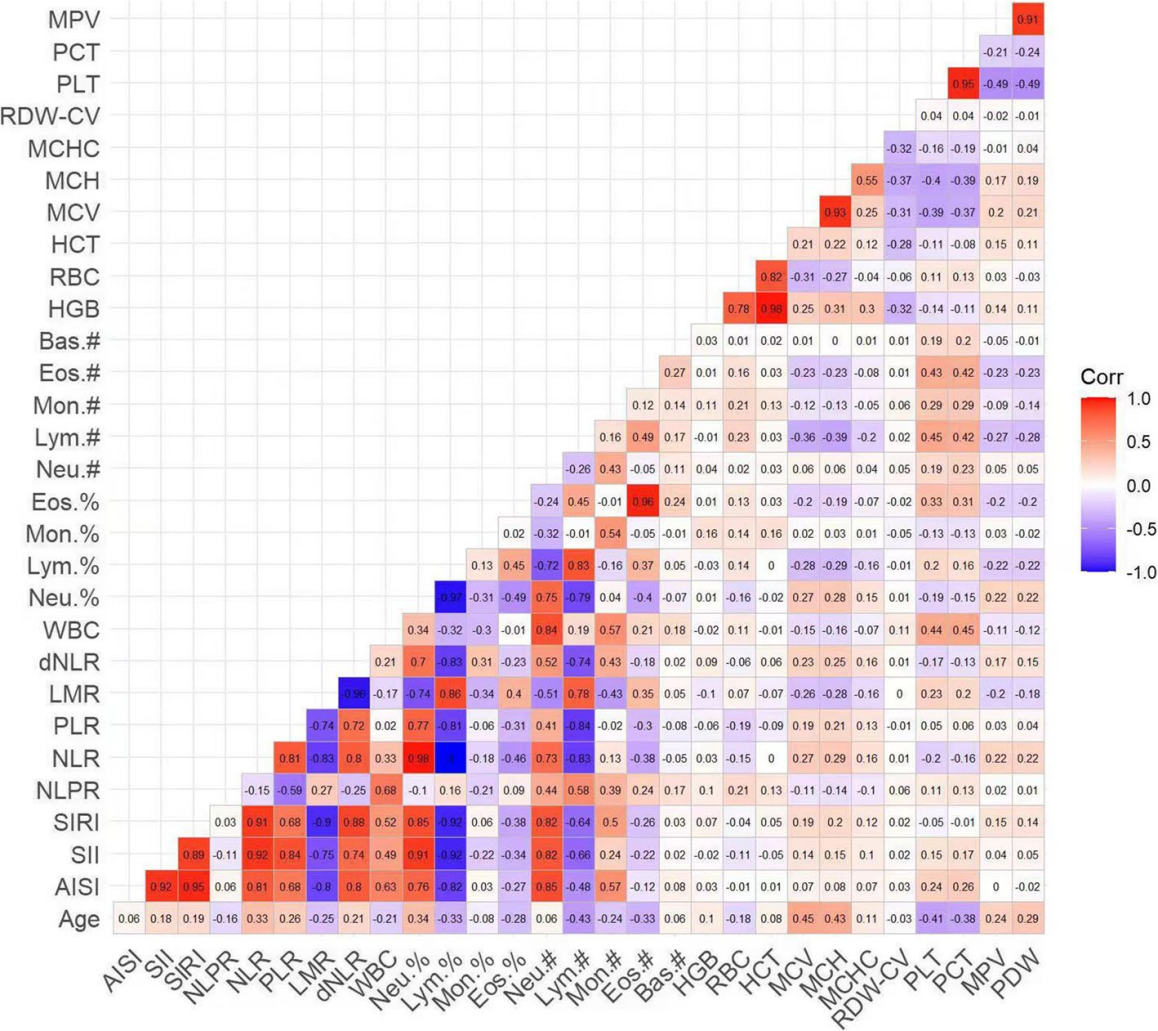
Study flow showing patients excluded from the study and the final cohort included in the study

Spearman’s correlation analysis method was employed to assess the relationship between various indicators and COVID-19. As illustrated in Fig. 2, significant positive correlations were identified between AISI and SII, SIRI, NLR, dNLR, Neu.# (with correlation coefficients of *r* = 0.92, *r* = 0.95, *r* = 0.81, *r* = 0.8, *r* = 0.85, respectively). Significant positive relationships were also found between SII and SIRI, NLR, PLR, Neu.%, Neu.# (with correlation coefficients of *r* = 0.89, *r* = 0.92, *r* = 0.84, *r* = 0.91, *r* = 0.82, respectively). Furthermore, SIRI showed significant positive correlations with NLR, dNLR, Neu.%, Neu.# (with correlation coefficients of *r *= 0.91, *r* = 0.88, *r* = 0.85, *r* = 0.82, respectively). NLR was significantly positively correlated with PLR, dNLR, Neu.% (with correlation coefficients of *r* = 0.81, *r* = 0.8, *r* = 0.98, respectively). Additionally, there was a significant positive relationship between LMR and Lym.% (*r* = 0.86), WBC and Neu.# (*r* = 0.84), Lym.% and Lym.# (*r* = 0.83), Eos.% and Eos.# (*r* = 0.96). Besides, HGB showed a significant positive correlation with HCT (*r* = 0.98), as did RBC with HCT (*r* = 0.82), MCV with MCH (*r* = 0.93), PLT with PCT (*r* = 0.95), and MPV with PDW (*r* = 0.91).

### Prediction model and performance

To predict the risk of COVID-19, we utilized the Deepwise & Beckman Coulter DxAI research platform (https://dxonline.deepwise.com/) for data analysis. Initially, 31 variables were significantly different between fever group and pneumonia group. Subsequently, a feature correlation analysis was performed on 31 indicators, eliminating features with a correlation coefficient lower than 0.9; these screened features were used for subsequent model training. To ensure the validity of the data, we conducted cross-validation with the dataset divided into 70% for training and 30% for validation.

**Fig. 3:**
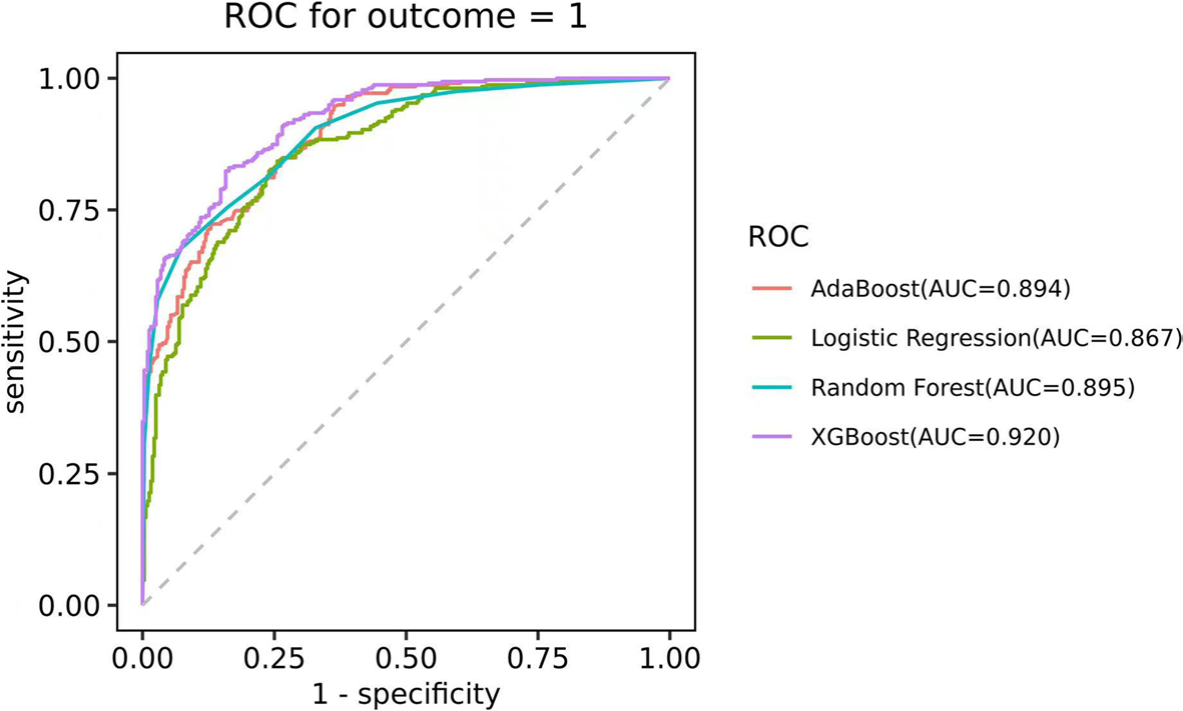
Receiver operating characteristic curves (ROC) showing the predictions of the four models: XGBoost, Random Forest, Logistic Regression and the AdaBoost

In this study, we attempted four common machine learning models: Adaboost, XGBoost, RandomForest, and LogisticRegression. Model performance was evaluated by the area under the receiver operating characteristic curve (AUC). The results indicated that the XGBoost model displayed optimal performance with an AUC of 0.920. Comparative models such as RandomForest, Adaboost, and Logistic Regression yielded AUCs of 0.895, 0.894, and 0.867, respectively, as detailed in Fig. 3.

**Fig. 4:**
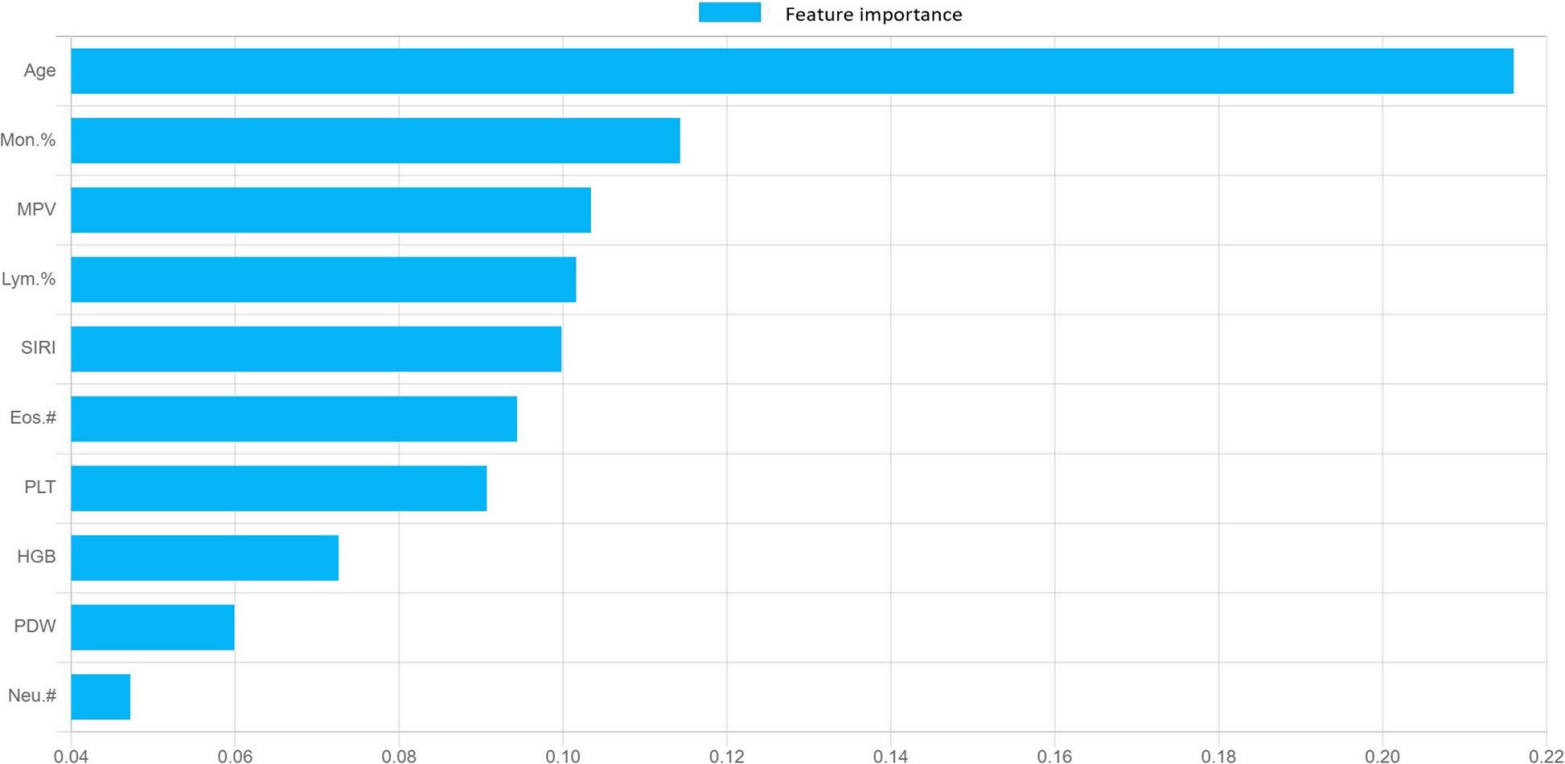
The top ten feature importance weights

**Fig. 5:**
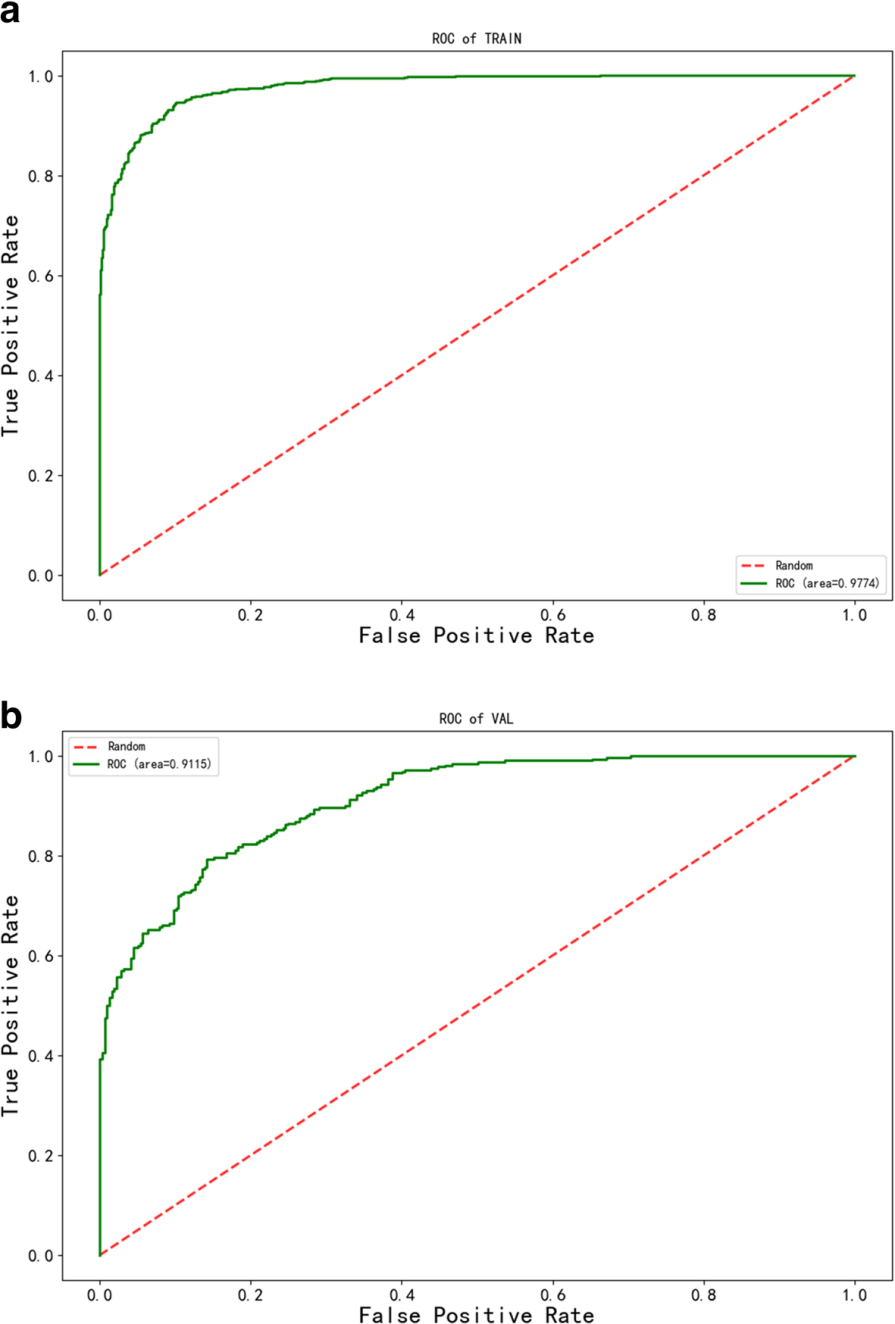
**a** AUC for the Training Sets. **b **AUC for the Validation Sets

Based on the order of feature weights from high to low, the top ten feature weights were selected to construct the XGBoost model, including Age, Mon.%, MPV, Lym.%, SIRI, Eos.#, PLT, HGB, PDW, and Neu.#. Among these, Age had the highest weight, as shown in Fig. 4. The model achieved an AUC of 0.977 on the training set, with a performance decrease on the validation set, yielding an AUC of 0.912. The performance of this model on the two subsets can be found in Table 2; Fig. 5a and b. In our external validation cohort, which included 513 COVID-19 patients (171 fever and 342 pneumonia), the predictive model showed notable efficacy. The ROC curve analysis yielded an AUC of 0.848, sensitivity of 0.719, specificity of 0.795, confirming the model’s effectiveness in predicting patient outcomes (Additional Files 1, Supplementary Table 1, Supplementary Fig. 1).

**Table 2:** Results of the confusion matrix for the training and validation sets

Variable	TRANI	VAL
Total Sample Size	1479	635
Positive Sample Size	739	318
AUC	0.977	0.912
Accuracy	0.914	0.819
Precision	0.915	0.835
Recall	0.912	0.796
F1 Score	0.913	0.815
Sensitivity	0.912	0.796
Specificity	0.915	0.842
PPV	0.915	0.835
NPV	0.912	0.804
AUC_CL	0.98[0.9716–0.9831]	0.91[0.8907–0.9322]

*AUC *Area Under the Curve *PPV *Positive Predictive Value, *NPV *Negative Predictive Value, *AUC_CL *Confidence Limits for AUC

**Fig. 6:**
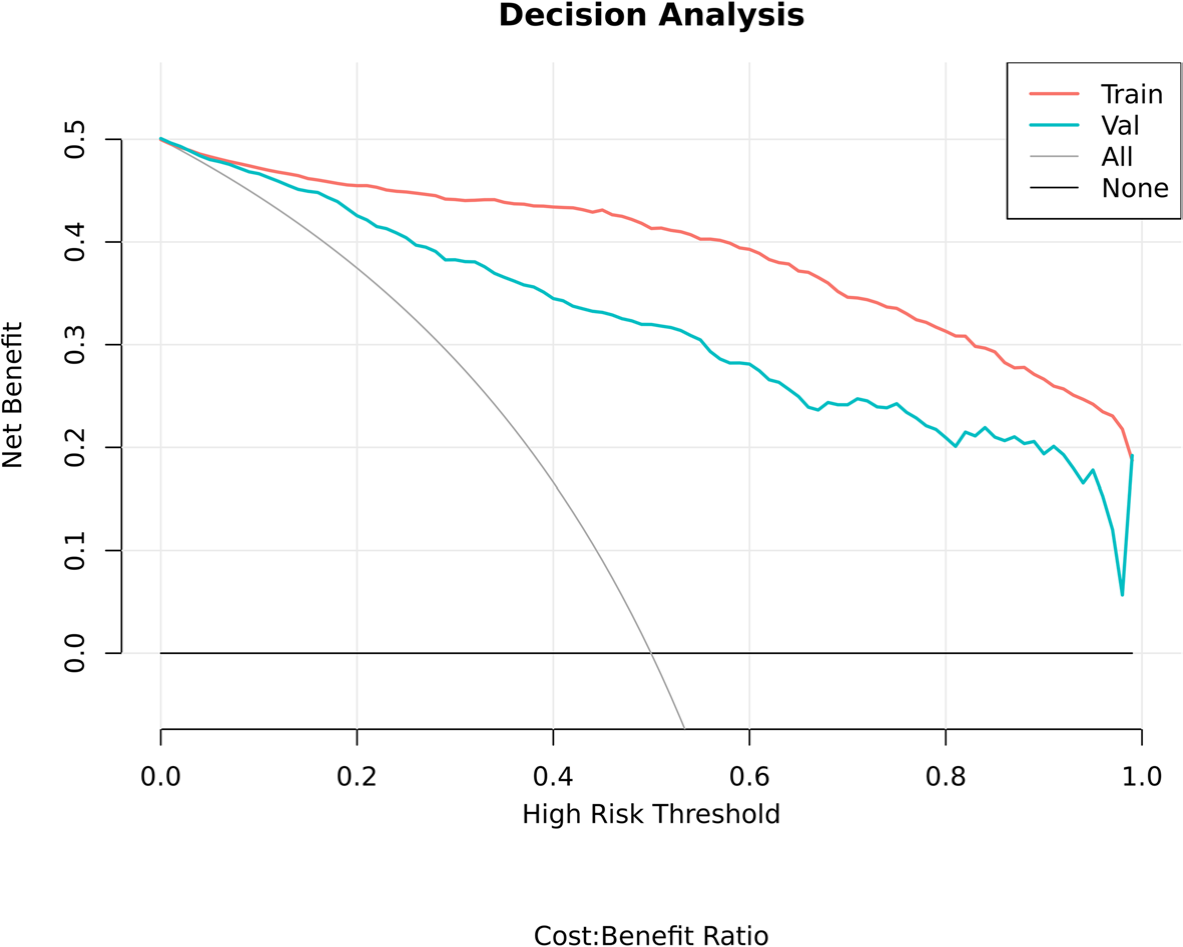
Decision Curve Analysis (DCA)

**Fig. 7:**
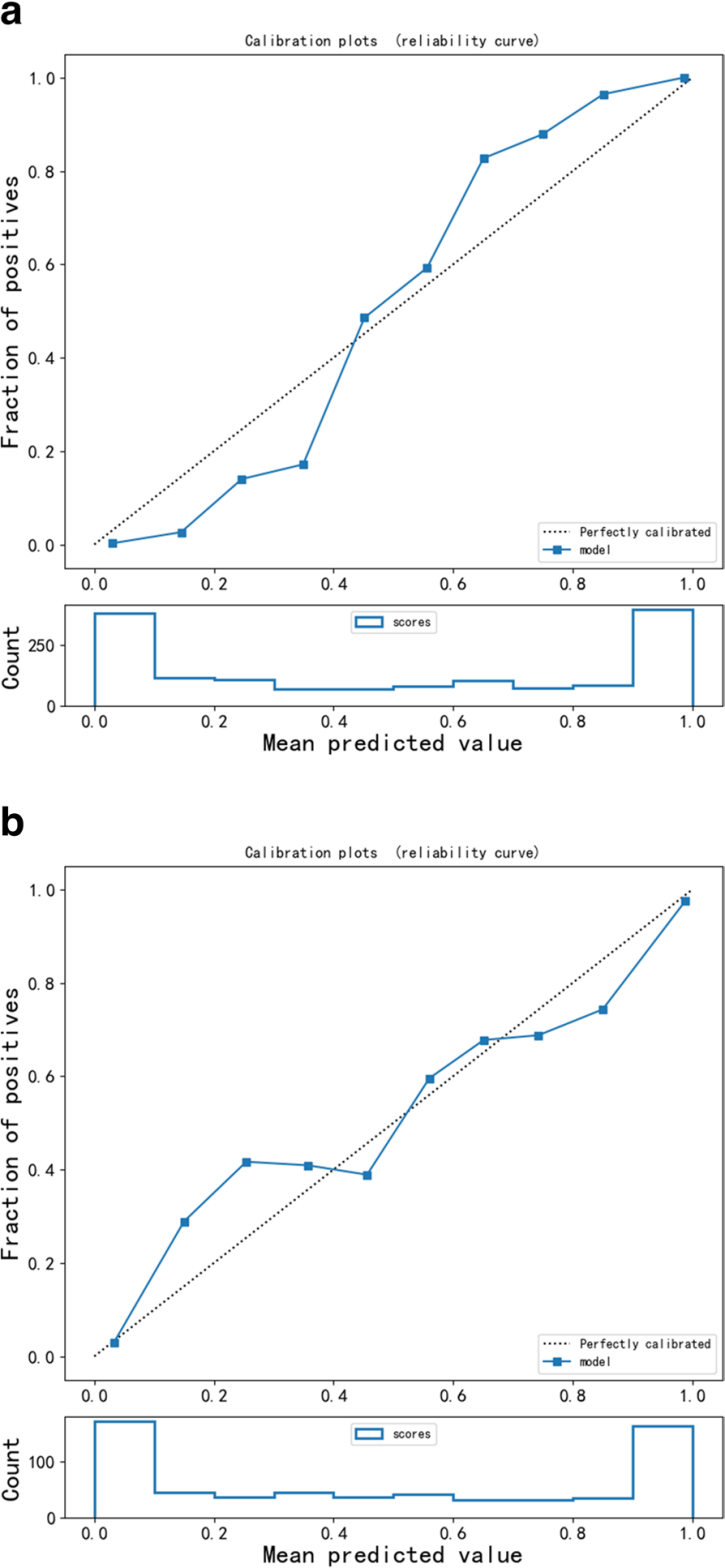
**a** Calibration Curveor the Training Sets. **b **Calibration Curveor the Training Sets

**Fig. 8:**
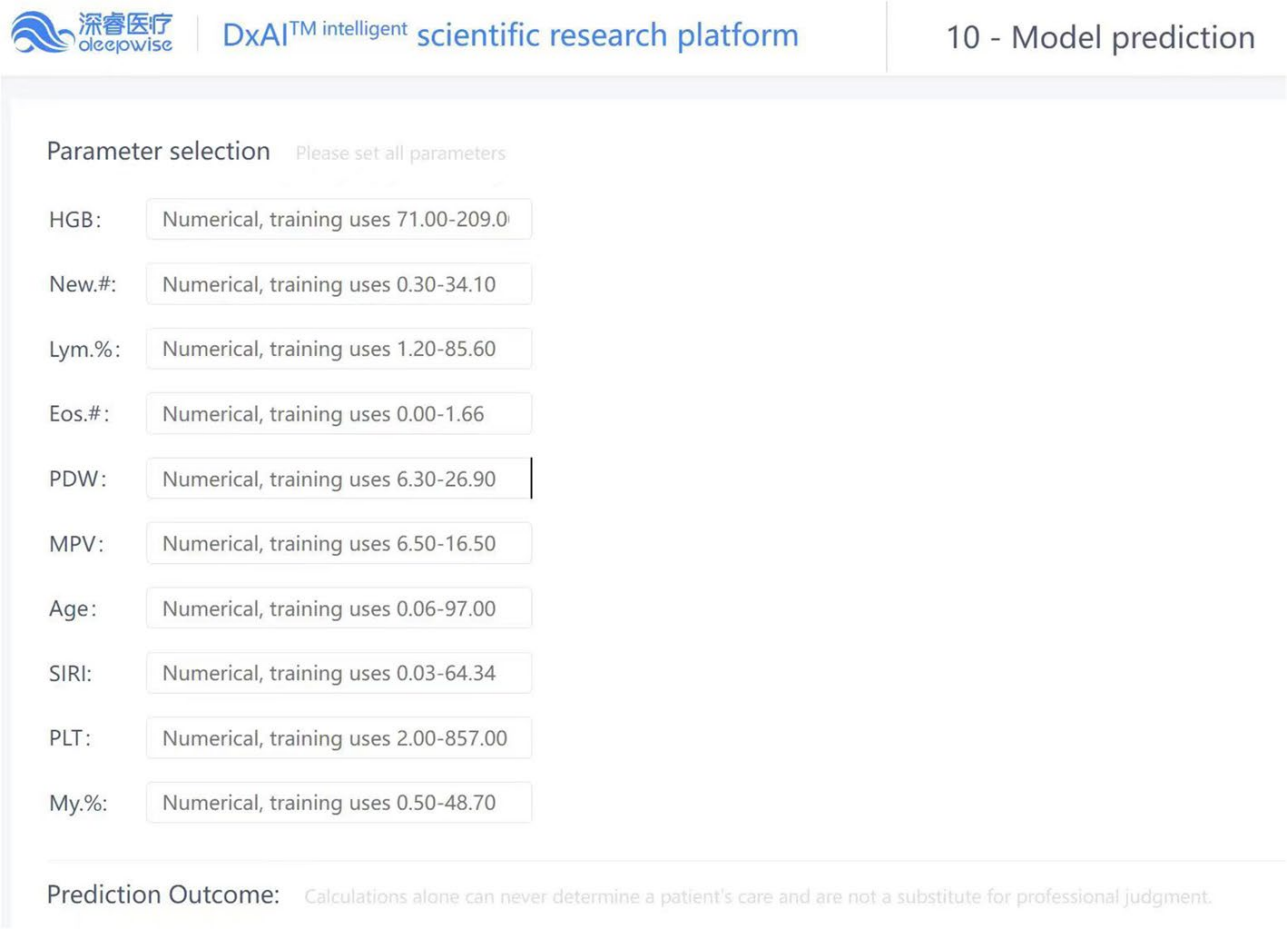
The visualization of the prediction model through Deepwise and Beckman Coulter DxAI platform. The Supplementary table 1 and figure 1 are located in file 1

 We rigorously evaluated the clinical applicability and potential benefits of the predictive model using Decision Curve Analysis (DCA). As shown in Fig. 6, this model exhibits significant advantages whether on the training set or the validation set. Further, as revealed by the calibration curve in Fig. 7a and b, the higher the consistency between the predicted and observed probabilities, the closer the calibration curve is to the 45-degree line, suggesting our model possesses a strong calibration effect. A webpage tool is displayed online through the Deepwise and Beckman Coulter DxAI platform, which generates predictive models based on the current algorithm, and can predict the risk of a positive result by setting parameters, as shown in Fig. 8. After inputting the CBC parameters, the patient could be discriminated as fever or pneumonia group with calculated probability. (https://dxonline.deepwise.com/prediction/index.html?baseUrl=%2Fapi%2 F&id=30759&topicName=undefined&from=share&platformType=wisdom).

## Discussion

In this study, following specific inclusion and exclusion criteria, a comprehensive retrospective analysis was conducted on 2114 COVID-19 patients. Based on their clinical features, patients were categorized into two groups: the fever group (*n* = 1057) and the pneumonia group (*n* = 1057). It was observed that the patients in the pneumonia group were older compared to those in the fever group. This could be attributed to the natural immunosenescence that accompanies aging, leading to a decreased immune response and rendering the immune system less effective in identifying and eliminating the virus, consequently increasing susceptibility to COVID-19. These findings align with the research conducted by Petter Brodin and colleagues, who identified various factors influencing the susceptibility and severity of COVID-19 infection and emphasized the crucial role of age [10]. This observation further validates the outcomes of our study.

In our laboratory analysis of hematological parameters, out of the 33 indicators evaluated, except for Hs-CRP and Bas.%, 31 indicators including WBC, Lym.%, Eos.%, Lym.#, Eos.#, PLT, PCT, Neu.%, Mon.%, Neu.#, Mon.#, HGB, RBC, HCT, MCV, MCH, MCHC, MPV, and PDW showed statistically significant differences between the two groups. Furthermore, these 31 indicators were subjected to collinearity analysis, and those with correlation coefficients exceeding 0.9 were excluded, resulting in the creation of four models (Adaboost, XGBoost, RandomForest, LogisticRegression) based on the remaining indicators. Among these, the XGBoost model demonstrated the best performance in terms of AUC, and it automatically selected the key feature variables, assigning corresponding weights to them.

The XGBoost algorithm has garnered substantial attention from researchers and has been extensively explored for application in predicting various diseases, including, but not limited to, forecasting and classifying heart diseases [11], establishing diagnostic models for breast cancer [12], staging liver cancer [13], optimizing the dosage of immunosuppressive drugs in kidney transplant patients [14], and analyzing survival factors influencing early mortality in colorectal cancer patients [15]. The XGBoost algorithm, a machine learning method based on gradient boosting trees, is renowned for its efficiency, flexibility, and scalability. It leverages abundant data and features to construct intricate nonlinear models, capturing risk factors and potential interactions contributing to the development of diseases. By integrating multifaceted data, including clinical data, radiographic data, and laboratory data, the XGBoost algorithm can establish predictive models that offer physicians accurate judgement and decision support, thereby enhancing therapeutic outcomes and mitigating adverse reactions [16, 17].

Weights are numerical parameters that signify the importance of different features or inputs within a model. They are learned and adjusted through the training data to enhance the model’s ability to fit and predict the data accurately. In the realm of clinical diseases, researching weights in relation to disease relevancy has become a topical issue. Gopi Battineni et al. diagnosed chronic diseases by analyzing weights within machine learning models, demonstrating high utility [18]. Guided by the importance of feature variables, we selected the top ten weights (Age, Mon.%, MPV, Lym.%, SIRI, Eos.#, PLT, HGB, PDW, and Neu.#) and rebuilt the XGBoost model, undergoing training and validation processes. Compared with the model constructed using 31 indicators, the model based on these ten indicators performed superiorly, exhibiting an AUC of 0.977 in the training set and an AUC of 0.912 in the validation subset. This presents high accuracy and stability in predicting COVID-19.

In the predictive model for COVID-19, the weight of the age factor prominently stands out, surpassing other variables in terms of its influence. Numerous studies corroborate that age undeniably serves as one of the crucial factors impacting susceptibility and severity of COVID-19 [19]. As age advances, the risk of infection correspondingly escalates, potentially attributable to the decline in immune function and the presence of other latent health issues. Research conducted by Liu and others compared the age distribution of SARS-CoV-2 infection and mortality rates in China, Italy, and South Korea, revealing a substantial number of fatalities among individuals over the age of 60 in these regions [20]. Hence, in predicting and managing COVID-19, an accurate assessment and substantial consideration of the age factor play a pivotal role in formulating effective prevention and treatment strategies.

Coronavirus Disease 2019 (COVID-19), an acute respiratory infection instigated by the novel coronavirus, implicates aberrations in the hematological system throughout its pathological process. This research unveils that through weight analysis, standard hematological parameters have gained substantial prominence in assessing predictive models for COVID-19. These weights reflect the impact degree of each feature variable on the predictive outcome. For the predictive model of COVID-19, the significance of these standard hematological parameters is indispensable.

The significance of monocytes in the predictive model for COVID-19 is underscored by their weight in the model, highlighting their crucial role in forecasting outcomes. Monocytes play a pivotal role in immune responses, engaging in both antiviral and anti-inflammatory processes. Following SARS-CoV-2 infection, substantial alterations occur in the phenotype and function of monocytes, which tightly correlate with the severity of the patient’s condition. Specifically, using a computational technique referred to as “virus tracking,” Pierre et al. conducted single-cell RNA sequencing on bronchoalveolar lavage fluid samples from patients with severe and mild COVID-19. Their findings revealed a concomitant infection of human metapneumovirus within monocytes of severe patients, particularly pronounced in those monocytes affected by interferon signaling [21]. These insights further substantiate the importance of monocytes in the COVID-19 predictive model, providing crucial clues to comprehend their mechanistic roles and disease progression.

Platelets, a type of blood cell, primarily contribute to blood coagulation and hemostasis, also demonstrating significant importance within the COVID-19 predictive model. Existing research indicates that SARS-CoV-2 may interact with platelets, thereby inciting an increase in platelet activation and aggregation [22]. This activation of platelets may correlate with the incidence of inflammatory responses and thrombus formation. Concurrently, anomalies in platelet counts may emerge in COVID-19 patients, with some presenting thrombocytopenia [23]. As crucial actors in immune response and inflammation, platelets can influence the body’s antiviral and anti-inflammatory responses. Hui Liu and colleagues have constructed a risk scoring model based on routine blood examination parameters, named the PAWNN score. Incorporating platelets and related data, this score accurately predicts the mortality risk of hospitalized COVID-19 patients and allows for dynamic monitoring throughout the hospital stay. Therefore, the inclusion of platelets in COVID-19 predictive models to assess their contribution and importance in prediction outcomes bears significant implications [24].

The role of lymphocytes in COVID-19 predictive models is significant. As a crucial category of immune cells, lymphocytes play an essential role in combating pathogen infection and maintaining immune homeostasis. In the process of SARS-CoV-2 infection, research has demonstrated that a reduction in lymphocyte counts correlates closely with disease severity, potentially associated with vascular homeostasis imbalance and immune cell dysfunction triggered by a cytokine storm [25].

The SARS-CoV-2 virus primarily gains entry into host cells and instigates infection by binding to the ACE2 receptor on host cells via its spike protein. Although initially thought to predominantly affect the respiratory system, increasing research suggests that it not only impacts the lungs but also potentially has ramifications on the heart and hematologic system. The virus achieves this by disrupting the binding of the spike protein with the heme in hemoglobin, thereby depriving it of its iron atoms, rendering it incapable of carrying oxygen, and damaging the hemoglobin [26]. Consequently, a state of hypoxia ensues within the human body, leading to symptoms such as dyspnea, cyanosis, and organ damage [27]. We discerned a notable weightage of neutrophils in the COVID-19 prediction model; these cells are critical elements of innate immunity and participate in the process of combating pathogen infections. Their presence is related to inflammation, cytokine storms, and the prognosis of critically ill patients [28, 29]. By conducting weight analysis, we find these features in routine blood tests to be of significant relevance in the COVID-19 prediction model. This discovery provides useful references for our in-depth research and further clinical applications.

However, XGBoost has limitations that need to be considered. Firstly, it performs better with larger training samples and abundant computational resources, but COVID-19 research often involves incomplete, imbalanced, and inconsistent data. Secondly, as COVID-19 is a rapidly evolving disease, relying solely on existing data may not capture real-time changes. XGBoost’s reliance on stable data distributions may affect its accuracy and stability in dynamic situations like COVID-19. Additionally, the dynamic nature of COVID-19 data limits XGBoost’s applicability for prediction. Therefore, combining other methods and techniques with XGBoost is essential to enhance its specificity and sensitivity in analyzing and predicting COVID-19-related issues.

In summary, our investigation reveals that the fusion of complete blood count (CBC) parameters with advanced machine learning techniques offers a powerful approach for the prediction of COVID-19. Our model, which prioritizes CBC as the cornerstone feature, stands out with an AUC of 0.920, underscoring a substantial leap in sensitivity and specificity for the detection of COVID-19 compared to existing models. For instance, our findings suggest that our model outperforms an integrated model using variance analysis coupled with LASSO and Boruta feature selection methods, which reported an AUC of 0.910 [30].

Furthermore, our results advocate for the incorporation of the XGBoost algorithm within the tapestry of current clinical workflows. Utilizing the model’s output as a supplementary aid in diagnostic protocols has the potential to streamline the prioritization process for PCR testing, fostering a more efficient clinical decision-making process. Such a model can serve as a cogent decision-support tool, interfacing seamlessly with hospital information systems to provide timely and accurate assessments for COVID-19 diagnosis and prognosis. To further improve the model’s real-world applicability and more portable for clinical use, the online application of the model utilized CBC biomarkers and could greatly improve the efficiency and coverage of COVID-19 diagnosis.

Nevertheless, while CBC biomarkers serve as potent predictors within our model, we emphasize the necessity of their contextual interpretation in conjunction with other clinical and imaging data. This holistic approach enriches the model’s precision and reliability. The implementation of such a comprehensive predictive model holds the promise of bolstering clinicians’ capabilities in navigating the pandemic’s challenges, optimizing screening processes, and tailoring patient-specific therapeutic strategies, ultimately aspiring to refine the overall management of COVID-19.

### Limitations

Our study, while offering substantial insights into predicting COVID-19, also bears some limitations. Although CBC indicators have shown significant advantages in predicting COVID-19, the incorporation of these indicators with other clinical information, including medical history, symptoms, and imaging findings, is essential to ensure the enhanced accuracy and reliability of the models. In the external validation cohort, the model also demonstrated favorable prognostic performance with an AUC of 0.848. Recognizing that our research is derived from a single hospital, we plan to expand our data collection to multiple hospitals in the future, which we believe will strengthen our findings and increase the robustness of our model. Additionally, we aim to enrich our model by incorporating more varied types of medical information, enhancing its predictive capacity.

Moreover, our study primarily used a retrospective analysis, which can introduce bias. Therefore, additional prospective studies with larger sample sizes are needed to validate our findings. Our study’s cohort was relatively homogenous, so future studies should aim to validate these models across diverse populations to account for potential confounding factors such as ethnicity, pre-existing conditions, and socio-economic status. Further research could also focus on the development of comprehensive and interpretable models that integrate data from multiple sources, including genomics and proteomics, thereby enhancing the predictive power and clinical utility of these models in the diagnosis and management of COVID-19. Although the developed model established good predictive power in both cohorts, our research population was from a single center. Further studies should include data from more hospitals and other populations to make our findings even stronger and our model more reliable. We also want to include more types of medical information to make our predictions better.

## Conclusion

The combination of complete blood count (CBC) and machine learning models shows promising potential in predicting COVID-19. By analyzing CBC indicators such as white blood cell count and lymphocyte ratio and utilizing the predictive capabilities of machine learning models, early diagnosis and risk assessment for COVID-19 can be provided. This approach has the ability to assist clinicians in epidemic monitoring, screening, and making personalized treatment decisions, ultimately improving diagnostic efficiency and prognosis assessment of COVID-19.

### Supplementary Information

**Additional file 1:** **Supplementary Table 1. **Results of the Confusion Matrix for the external validation cohort. **Supplementary Figure 1. **ROC Curve Analysis for External Validation Cohort. Receiver Operating Characteristic (ROC) curve for the predictive model tested on the external validation cohort of 513 COVID-19 patients, illustrating the model's diagnostic performance. The area under the curve (AUC) is 0.848, indicating a high level of accuracy in discriminating between patient outcomes.

## Data Availability

All data generated or analyzed during this study are included in this published article and its supplementary information files.

## Abbreviations

AISI: Aggregate index of systemic inflammation
dNLR: Derived neutrophil to lymphocyte ratio
MLR: Monocyte to lymphocyte ratio
MPR: Mean platelet volume to platelet ratio
NLR: Neutrophil to lymphocyte ratio
NLPR: Neutrophil to lymphocyte ╳ platelet ratio
PLR: Platelet to lymphocyte ratio
SII: Systemic immune-inflammation index
SIRI: Systemic inflammation response index
WBC: White blood cells
HGB: Haemoglobin
HCT: Hematocrit
MCV: Mean corpuscular volume
MCH: Mean corpuscular hemoglobin
MCHC: Mean corpuscular hemoglobin concentration
RDW: Red blood cell distribution width
PLT: Platelet
PCT: Thrombocytocrit
MPV: Mean platelet volume
PDW: Platelet distribution width
hs-CRP: Hypersensitive C-reactive protein
ROC: Receiver operating characteristic curves
AUC: Area Under the Curve
PPV: Positive Predictive Value
NPV: Negative Predictive Value
AUC_CL: Confidence Limits for AUC
DCA: Decision Curve Analysis
