# Supplementary material for: Online COVID-19 diagnosis prediction using complete blood count: an innovative tool for public health
Source: BMC Public Health. 2023 Dec 19;23:2536. doi: 10.1186/s12889-023-17477-8 (PMC10729447; doi:10.1186/s12889-023-17477-8)
Supplement: Supplementary file 1 — Additional file 1: Supplementary Table 1. Results of the Confusion Matrix for the external validation cohort. Supplementary Figure 1. ROC Curve Analysis for External Validation Cohort. Receiver Operating Characteristic (ROC) curve for the predictive model tested on the external validation cohort of 513 COVID-19 patients, illustrating the model's diagnostic performance. The area under the curve (AUC) is 0.848, indicating a high level of accuracy in discriminating between patient outcomes. [file 12889_2023_17477_MOESM1_ESM.docx]

# ADDITIONAL FILE #1:

# Supplementary Materials

| **Supplementary Table 1** |  |
| --- | --- |
| Results of the Confusion Matrix for the external validation cohort | |
|  | TEST |
| Total Sample Size | 513 |
| Positive Sample Size | 342 |
| AUC | 0.848 |
| Accuracy | 0.745 |
| Precision | 0.875 |
| Recall | 0.719 |
| F1 Score | 0.790 |
| Sensitivity | 0.719 |
| Specificity | 0.795 |
| PPV | 0.875 |
| NPV | 0.586 |
| AUC_CL | 0.850[0.8121-0.8844] |
| AUC: Area Under the Curve; PPV: Positive Predictive Value; NPV: Negative Predictive Value; AUC_CL: Confidence Limits for AUC | |


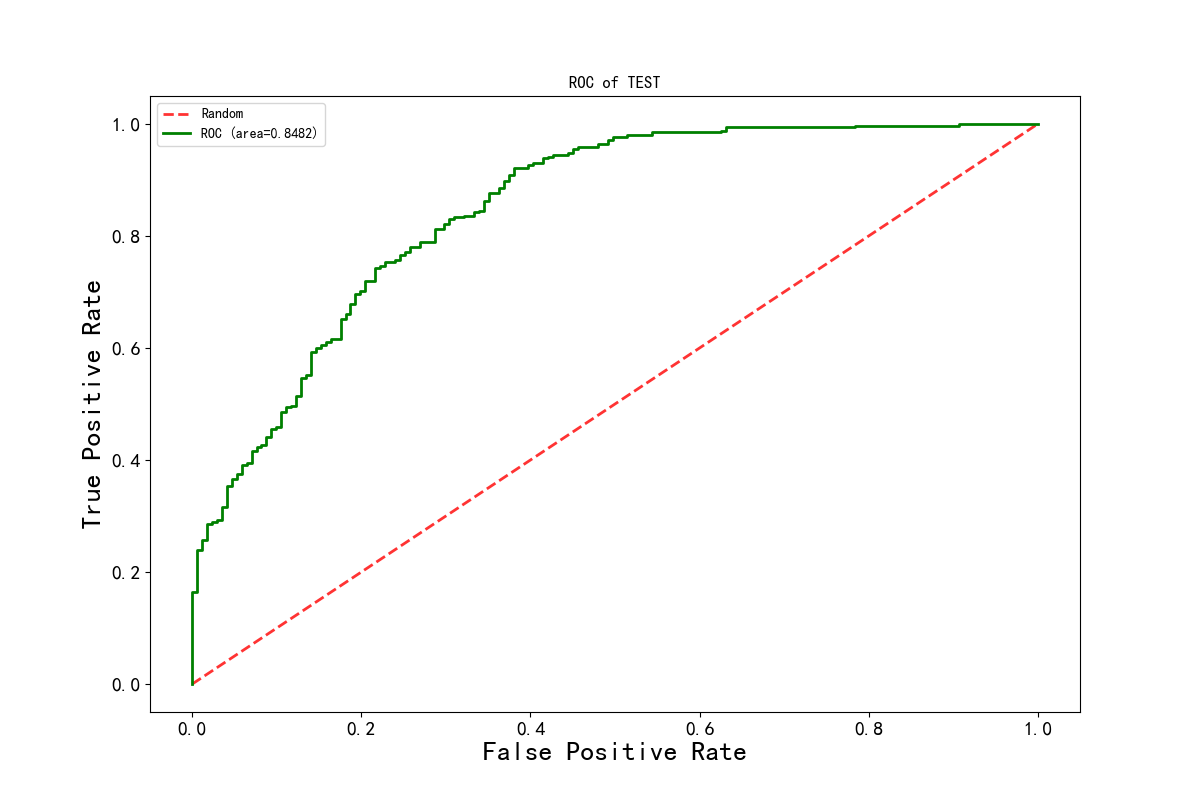


**Supplementary Figure 1** ROC Curve Analysis for External Validation Cohort

Receiver Operating Characteristic (ROC) curve for the predictive model tested on the external validation cohort of 513 COVID-19 patients, illustrating the model's diagnostic performance. The area under the curve (AUC) is 0.848, indicating a high level of accuracy in discriminating between patient outcomes.
